# Medication Trials for Hyperphagia and Food-Related Behaviors in Prader–Willi Syndrome

**DOI:** 10.3390/diseases3020078

**Published:** 2015-06-03

**Authors:** Jennifer L. Miller, Theresa V. Strong, Janalee Heinemann

**Affiliations:** 1 Department of Pediatrics, Division of Endocrinology, University of Florida College of Medicine, 32607 Gainesville, FL, USA; 2 Department of Medicine, University of Alabama at Birmingham, 35294 Birmingham, AL, USA; E-Mail: theresa.strong@fpwr.org; 3 Foundation for Prader-Willi Research, 91789 Walnut, CA, USA; 4 Prader-Willi syndrome Association USA, 34238 Sarasota, FL, USA; E-Mail: JHeinemann@pwsausa.org

**Keywords:** Prader-Willi syndrome, hyperphagia, drug trials, obesity

## Abstract

Prader-Willi syndrome (PWS) is a neurodevelopmental disorder caused by the absence of paternally expressed, imprinted genes on chromosome 15q11-13. Individuals with PWS characteristically have poor feeding and lack of appetite in infancy, followed by the development of weight gain and then uncontrolled appetite and lack of satiety, sometime after the age of two. The overwhelming drive to eat is coupled with reduced energy expenditure and decreased caloric requirements, thus, individuals with PWS will become severely obese unless their food intake is strictly controlled. The mechanisms underlying hyperphagia in PWS remain incompletely understood, and to date no drugs have proven effective in controlling appetite. However, clinical trials have started for several medications, which may provide therapeutic options for those with PWS. These medication trials may also provide insight into potential treatments for obesity in the general population. Ideally, these treatments will help alleviate the complex metabolic issues that are part of this syndrome.

## 1. Introduction

Prader-Willi syndrome (PWS) is a complex multisystem syndrome that occurs with a frequency of approximately 1/15,000 to 1/30,000 and has significant morbidity, including central hypotonia, developmental disabilities, behavioral issues, growth hormone deficiency and hypogonadism, and obesity compounded by a centrally driven excessive appetite (hyperphagia). It is the most commonly known genetic cause of obesity. PWS results from the loss of expression of paternally derived genes by a variety of mechanisms, which include large deletions (70%–75%), maternal uniparental disomy (UPD, 20%–30%), and imprinting defects (2%–5%) [1,2,3]. The body composition of individuals with PWS is significantly different from that of individuals in the general population. Patients with PWS have decreased lean body mass and increased adiposity that is apparent throughout life.

PWS is a spectrum disorder, which affects multiple body systems. The underlying pathophysiological basis of the PWS phenotype remains unclear. Current therapies for PWS show limited efficacy and are directed to various symptoms associated with the disease process. Growth hormone (GH) deficiency is present in almost all children, and many adults, with PWS. Growth hormone replacement therapy is standard of care for children with PWS and increasingly prescribed in infants and adults. GH replacement therapy is effective in increasing height, improving body composition, increasing bone mineral density, improving exercise endurance and quality of life, and favorably impacting development and behavior [4]. However, it does not affect hyperphagia. Intellectual disability and neuropsychiatric issues are present to some degree in all individuals with PWS [5,6]. Intellectual quotient (IQ) is generally in the mild range, with some individuals demonstrating low normal IQ and others exhibiting moderate intellectual disability. Behavioral difficulties include cognitive rigidity, emotional liability, and obsessive-compulsive type symptoms, with autistic symptomatology also frequently reported [7]. Additional clinical complications frequently found in PWS include excessive daytime sleepiness, scoliosis, osteopenia/osteoporosis, decreased gastrointestinal motility, sleep disturbances, and reduced pain sensitivity [1].

Individuals with PWS experience a characteristic change in food intake over time. Infants with PWS typically exhibit hypotonia, lethargy, a weak or discoordinated suck and swallow mechanism, and failure to thrive [8]. Infants with PWS do not exhibit signs of hunger and typically require force-feeding via gastrostomy tube, nasogastric tube, or cross-cut nipples on the bottle. Hypotonia in infants with PWS slowly resolves over time, although adults with PWS continue to exhibit some muscle hypotonia and weakness throughout their lifetime. Nutritional support is critical to optimize development in infancy, but feeding typically improves in young children. Excessive weight gain begins typically before the appetite increases, and then hyperphagia commences sometime during childhood. As adolescents and adults, all individuals with PWS will become morbidly obese if strict environmental controls are not implemented.

The underlying molecular mechanisms that contribute to excessive weight gain and hyperphagia in PWS remain incompletely understood. Most gut-related appetite regulating hormones are normally expressed in PWS, with the most notable exception being ghrelin, a growth hormone secretagogue that is high in fasting states and decreases with eating. Highly elevated levels of ghrelin have been identified in individuals with PWS. Ghrelin levels decrease after intake of a meal, as is normal, but still remain comparatively high. Thus, the relationship between hyperphagia and ghrelin remains unclear, and it has been proposed that the hyperghrelinemia may be the result of hypoinsulinemia [9]. Leptin levels are appropriately elevated for degree of obesity, indicating that leptin deficiency is not a cause of the appetite issues.

Appetite and satiety issues are regulated by a complex interplay between gut hormones and hypothalamic neuropeptides. With regards to the neuropeptides that regulate hunger/satiety circuits, Swaab and colleagues [10] reported a deficit in the oxytocin (OT)-producing neurons of the paraventricular nucleus (PVN) in the brain of these patients and Bittel and colleagues [11] reported decreased oxytocin receptor gene function in PWS. OT is a well-known satiety hormone that is also involved in establishing and maintaining social standards. It is thought that perhaps the low levels of OT in the brain of individuals with PWS may contribute to the hyperphagia. Other studies have suggested that the hyperphagia may be caused by abnormalities in another hypothalamic hormone, hypocretin/orexin. Orexin regulates the sleep/wake cycle, metabolism, and appetite, and so is a viable candidate hormone to explain several of the clinical findings in PWS. It is known that the sleep/wake cycle is abnormal in individuals with PWS, many of whom suffer from excessive daytime sleepiness and narcolepsy, a disorder caused by abnormally low hypocretin/orexin. Within the PWS region on Chromosome 15, the SNORD116 host gene transcript has been reported to regulate expression of diurnal and circadian genes in the brain, suggesting a potential role in energy expenditure and hyperphagia in PWS [12]. However, despite considerable investigation, at this time there are no consistently identified hormone abnormalities to fully explain the hyperphagia in PWS, and the metabolic correlates of hyperphagia are still unclear.

To date, no medication has proven effective in regulating appetite in PWS. Bariatric surgery is not accompanied by a decrease in appetite that occurs in typical individuals and is contraindicated in this population [13]. Other medications evaluated to date in PWS have proven ineffective [14,15] or have been associated with unacceptable side effects [16].

## 2. Hyperphagia and Quality of Life Considerations

The inability to control food intake is often the biggest obstacle keeping those with PWS from living independently, participating in normal social events, and working in unrestricted settings. The lack of satiety associated with PWS has chronic consequences (morbid obesity) as well as potentially acute consequences. Without supervision, individuals with PWS will die prematurely as a result choking (especially during episodes of voracious eating), stomach rupture or tissue necrosis, or from complications caused by morbid obesity [17,18]. In addition to limiting living arrangements, opportunities for community engagement and social activities are highly constrained by food access issues. Similarly, given need for 24/7 supervision regarding food, hyperphagia strictly limits employment opportunities, even for those individuals with higher intellectual function. Finally, extreme behaviors such as anxiety, aggression, perseveration, are common in PWS, and frequently revolve around food issues. Thus, this is a population to whom an effective therapy for appetite and obesity has the potential for a profound positive effect on lifestyle opportunities and quality of life.

### 2.1. Support Groups

Families and individuals with PWS are fortunate to have significant community support in the form of parent-run foundations. These groups—the Prader-Willi Syndrome Association of the United States (PWSA-USA), the Foundation for Prader-Willi Research (FPWR), and the International Prader-Willi Syndrome Organisation (IPWSO)—have lobbied for years to try to get pharmaceutical companies interested in PWS as a model for obesity, with the idea that if the appetite in PWS can be treated, the appetite of anyone with obesity can be treated. The motto that “PWS is a window into the house of obesity” has finally begun to resonate with pharmaceutical companies, and currently there are several medication trials which are ongoing, with more yet to come.

### 2.2. Medication Trials

Current medication trials in PWS are summarized in Table 1. The first company to express an interest in research for treatment of hyperphagia in PWS was Zafgen, which manufactures a medication called beloranib. Beloranib is a methionine aminopeptidase 2 (MetAP2) inhibitor that decreases fat biosynthesis and enhances fat oxidation and lipolysis [19]. In studies of animal models with diet-induced obesity or hypothalamic obesity, beloranib resulted in substantial and persistent weight loss, decreased appetite, and improvements in metabolic and inflammatory biomarkers [20]. Additionally, in studies of adults with exogenous obesity, beloranib caused substantial weight loss and a significant reduction in hunger [21]. Therefore, the benefits of this medication seem uniquely suited to the needs of individuals with PWS. The phase 2 trial of beloranib was completed in 2013 and demonstrated a good safety and efficacy profile in this population. Phase 3 trials are currently ongoing.

Rhythm Pharmaceuticals has developed a medication called RM-493 which is a melanocortin 4 receptor (MC4R) agonist for trial in individuals with PWS. The basis for this medication is that the pro-orexigenic pathways in the hypothalamus, which are apparently normally expressed in the brains of individuals with PWS, can potentially be overcome by activation of the MC4R, thus controlling appetite and improving metabolism. RM-493, cardiovascularly safe and efficacious MC4R agonist, is a potent activator of the MC4R, thus making it a target for potential treatment of both the hyperphagia and low metabolism in individuals with PWS. Phase 2a trials are currently in progress to evaluate the safety and efficacy of this medication in PWS.

Oxytocin (OT) has been of interest as a potential treatment for PWS since the abnormalities in OT neurons were identified in 1995. Oxytocin is involved in establishing and maintaining social standards. Indeed, it has recently been shown in a double blind placebo study, that OT administration to adults with PWS significantly decreased depressive mood tendencies and tantrums while increasing trust in others, with data supporting a trend to decrease appetite with higher satiety [22]. Moreover, in a PWS knock-out mouse model for the *Magel2* gene, a single OT injection at 5 hours of life prevented early death observed in 50% of new-born mice by recovering a normal suck [23]. However, a study of adolescents and adults with PWS treated with OT twice daily failed to show any improvements in appetite, and there was even some worsening of behaviors [24]. This medication needs further investigation to determine if it is helpful in ameliorating the excessive appetite in PWS and if there is potentially an overdose effect that could worsen behaviors. Ferring Pharmaceuticals has developed an intranasal oxytocin analogue (Intranasal FE 992097), which exerts its effects on the OT receptors in the brain. Phase 2a studies have been completed on this medication in individuals with PWS.

**Table 1:** Current Medication Trials in Prader-Willi syndrome (PWS).

Medication	Company	Compound	Target Action	Study Phase	ClinicalTrials.gov Identifier
Beloranib	Zafgen	methionine aminopeptidase 2 (MetAP2) inhibitor	Decrease fat biosynthesis and enhance fat oxidation and lipolysis	Phase 3	NCT02179151
RM-493	Rhythm	melanocortin 4 receptor (MC4R) agonist	Activation of the MC4R to control pro-orexigenic pathways in the hypothalamus	Phase 2	NCT02311673
Intranasal Oxytocin	N/A	Oxytocin nasal spray	Bind oxytocin receptors to improve satiety and behaviors	Phase 2	NCT02013258
Intranasal FE 992097	Ferring	Oxytocin analog	Bind oxytocin receptors to decrease hyperphagia and behaviors	Phase 2 completed	NCT01968187
Diazoxide	Essentialis	K^+^-ATP channel agonist	Hyperpolarize hypothalamic neurons whose activity is otherwise impaired by a defective leptin signaling pathway	Phase 2	NCT02034071
AZP-531	Alize	unacylated ghrelin analog	Increase unacylated ghrelin levels to improve blood glucose levels and weight	Not started	Not yet listed
Exanatide/Liraglutide	AstraZeneca/Novo Nordisk	GLP-1 Receptor Agonists	Suppress appetite and to induce weight loss	Phase 2	NCT01444898/NCT01542242

Diazoxide (Essentialis) is a medication which has been used for many years for individuals with hyperinsulinism, so has a well-characterized safety profile. Diazoxide is a potent K^+^-ATP channel agonist which is hypothesized to potentially address many of the abnormalities observed in patients with Prader Willi Syndrome by hyperpolarizing hypothalamic neurons whose activity is otherwise impaired by a defective leptin signaling pathway. Phase 2 studies are almost completed in children with PWS.

Alize has developed an unacylated ghrelin analog (AZP-531) which has been shown in phase 1 studies to improve blood glucose levels and weight with once daily administration in individuals with obesity and type 2 diabetes. This company is preparing for phase 2 trials targeting treatment for individuals with PWS and those with type 2 diabetes.

Exanatide and Liraglutide are glucagon-like peptide-1 (GLP-1) receptor agonists which were originally developed for treatment of Type II Diabetes. This class of drugs has been shown to suppress appetite and to induce weight loss in both diabetic and non-diabetic individuals. A small 2011 placebo controlled crossover study reported that a single injection of exanatide improved satiety and lowered circulating levels of both glucose, insulin, PYY, and GLP-1 in both individuals with PWS and those with exogenous obesity [25]. Ghrelin levels and energy expenditure were not affected and there were minimal side effects in the PWS group. Two case reports of liraglutide for treatment of diabetes in individuals with PWS suggested improvement in hyperphagia during the treatment period [26,27]. These results warrant larger, longer-term studies. A 6-month study of exanatide was just completed in individuals with PWS. A significant concern with respect to the long term use of GLP-1 receptor agonists in the PWS population is that these medications delay stomach emptying. Delayed stomach emptying, decreased intestinal motility and gastroparesis are common findings in PWS, and pilot studies (unpublished) indicate that GLP-1 receptor agonists further exacerbate abnormally slow gut movement in PWS.

There are also some other FDA-approved medications which may potentially improve satiety or help ameliorate obesity in individuals with PWS, including lorcaserin, naltrexone HCl/bupropion HCl (Contrave^®^), and phenteramine/topiramate (qsymia^®^). There are also some devices in testing which may also improve appetite and behavior in individuals with PWS, including transcranial direct magnetic stimulation and vagal nerve stimulators.

## 3. Conclusions

This is a tremendous time of hope for potential treatment of the appetite issues in PWS. Given the fact that the great majority of patients are now diagnosed in infancy, the combination of early intervention and early growth hormone therapy, along with a medication to decrease the appetite issues could result in a much improved quality of life, and increased independence, for individuals with Prader-Willi syndrome.
